# Cost drivers associated with diffuse large B-cell lymphoma (DLBCL) in Japan: A structural equation model (SEM) analysis

**DOI:** 10.1371/journal.pone.0269169

**Published:** 2022-05-27

**Authors:** Saaya Tsutsué, Shinichi Makita, Jingbo Yi, Bruce Crawford

**Affiliations:** 1 Celgene K.K., a Bristol Myers Squibb Company, Tokyo, Japan; 2 National Cancer Center Hospital, Tokyo, Japan; 3 Syneos Health, Tokyo, Japan; Murdoch University, AUSTRALIA

## Abstract

Diffuse large B-cell lymphoma (DLBCL) is an aggressive non-Hodgkin’s lymphoma of increasing prevalence in Japan. However, patients with relapsed or refractory disease to first line treatment (rrDLBCL) have been found to shoulder greater economic burden and have poor survival with subsequent lines of therapy. The relative impact of individual patient attributes on total medical cost among patients with rrDLBCL receiving second or third line (2L/3L) therapy was assessed. Structural equation modelling was used to identify potential cost drivers of total medical costs incurred by treatment and procedures in a Japanese retrospective claims database. From the database, rrDLBCL patients on 2L or 3L of treatment were grouped into respective cohorts. The mean [median] (SD) total medical cost of care for the 2L cohort was 73,296.40 [58,223.11] (58,409.79) US dollars (USD) and 75,238.35 [60,477.31] (59,583.66) USD for the 3L cohort. The largest total effect on medical cost in both cohorts was length of hospital stay (LOS) (β: 0.750 [95%CI: 0.728, 0.772] vs β: 0.762 [95%CI: 0.729, 0.794]). Length of hospital stay and potential heart disease complications due to line of treatment were the primary drivers of total cost for patients who had received at least 2L or 3L therapy for rrDLBCL.

## Introduction

The incidence of aggressive non-Hodgkin lymphoma (NHL) has been increasing steadily in Japan. By 2008, NHL was responsible for 39.6% of all hematologic malignancies nationwide [1]. Diffuse large B-cell lymphoma (DLBCL) accounts for a large proportion of such lymphoid neoplasms in Japan (35.8%) and regional disease proportion varies between 25.7% to 39.5% [2]. The standard treatment for DLBCL is R-CHOP regimen (rituximab [R] + cyclophosphamide/doxorubicin/vincristine/prednisone) administered for 6–8 cycles. A United States (US) claims-based study found that 87.7% of DLBCL patients received combination therapies, and 69.7% had received R-CHOP [3]. A population-based cancer registry in Japan reported the 5-year overall survival for DLBCL patients to be 57% in 2003–2006, a 13% increase from 1993–1997 [4]. However, after stopping treatment, up to 50% of patients may become relapse or become refractory to further treatment [5].

Patients with relapsed or refractory (rrDLBCL) have poor prognosis and unstandardized treatment regimens during subsequent treatment lines [6]. A large study of pooled patient level data in the US demonstrated rrDLBCL patients had an overall response rate of 26% to further treatment and a median survival of 6.3 months [7]. Even after autologous stem cell transplantation (auto-SCT), median OS for rrDLBCL patients was 9.9 months [8]. While it is critical to understand not only the real-world course of treatment, but also the drivers of those medical costs for patients, there is a paucity of research on the economic burden of rrDLBCL in Japan.

Even with poor survival outcomes, the economic burden of DLBCL is high. The average DLBCL-related cost per patient per year in the first year of treatment was reported to be significantly higher for second line (2L) DLBCL patients (210,488 US dollars (USD)) compared to first line (1L) patients (25,044 USD) in the US [9]. A separate analysis of the economic burden for matched 1L and 2L DLBCL cohorts in the US highlighted clinical services as the main incremental cost drivers (outpatient (50%) and inpatient (36%) services) [10]. The relationship between the direct and indirect drivers of medical costs for rrDLBCL in Japan, as well as any intermediate effects, remain unclear.

In this study, structural equation modeling (SEM) was used to explore the relationship between patient characteristics, healthcare resource utilization (HCRU) and medical costs for rrDLBCL. Identifying drivers of medical cost may provide insights into how to reduce the economic burden for Japanese patients.

## Materials and methods

### Study design and study population

An administrative retrospective claims database (2008–2020) provided by Medical Data Vision Co., Ltd. (MDV; Tokyo, Japan) was used in this study. Covering approximately 23% of acute hospitals and 30 million patients, the MDV database is a large database of anonymized medical claims from over 400 acute care hospitals in Japan.

The identified patients had at least one DLBCL-related treatment claim between October 1, 2008 and June 30, 2019. The first treatment date was defined as the date of first DLBCL-related treatment (1L) during this period with the appropriate International Classification of Disease 10th revision (ICD-10) diagnosis code (C83.3x, C85.2x or receiptcode 8847286). Records must have had a 6-month lookback period from index date with at least 1 claim (for any disorder) as used in a previous database study [11]. Minimum follow-up period for inclusion was 12 months and patient records that did not have at least 2 claims (1 claim every 6-month period for any disorder) were excluded in order to capture sufficient cost for this study to conduct SEM. Remaining patients were included in further analysis if they had received either 2L or 3L during the identification period. Two separate cohorts (with overlapping patients) were analyzed; one for patients who initiated 2L and one for patients who initiated 3L. Index date was defined as the first administration of second line for the 2L cohort and third line for the 3L cohort. Database was downloaded on 5th Oct 2020.

### Patient characteristics

Patient demographics tabulated of which include gender, age, and age group. Clinical characteristics including year of index date, prior treatment regimen, potential complications due to treatment, duration of therapy (1L-3L), baseline Charlson Comorbidity Index (CCI) score with breakdowns of each comorbidities, including a modified index excluding diagnosis of DLBCL itself, were analyzed to describe the study cohorts.

Potential complications from 2L/3L treatment, including heart disease [12], kidney disease [13], and liver disease [14], were defined as new events after index date among those without these conditions during any prior lines of therapy. Prior or concurrent cancers during the look-back period were also assessed (C00-C96 except for C77-89, i.e. exclude secondary neoplasms and lymphomas). The average duration of each line of therapy was calculated (1L-3L) as months from the first treatment date to the last treatment date records. CCI scores were calculated using the look-back period (prior to start of 2L/3L treatment) based on the ICD-10 codes associated with the modified CCI [15].

DLBCL-related treatment was summarized for drugs received within ±30 days of first line treatment initiation so to also include patients in the middle of their treatment cycle. Subsequent line of treatment for all included patients were extracted up to 5L+. Treatment lines were grouped in a hierarchical order based on their regimen components: DeVIC-based (dexamethasone, etoposide, ifosfamide, carboplatin) with or without rituximab, R-CHASE-based (rituximab, cyclophosphamide, cytarabine, etoposide, dexamethasone), GDP-based (gemcitabine, dexamethasone, cisplatin) with or without rituximab, R-Bendamustine-based, R-EPOCH-based (rituximab, etoposide, prednisone, vincristine, cyclophosphamide, doxorubicin), R-ESHAP-based (rituximab, etoposide, cytarabine, cisplatin, methylprednisolone), ESHAP-based, R-ICE-based (rituximab, ifosfamide, carboplatin, etoposide), R-DHAP-based (rituximab, dexamethasone, cytarabine, cisplatin), other R-based, and other chemotherapy without rituximab. Lastly, patients receiving conditioning regimens before auto-SCT were also extracted (including MINE, LEED, MCEC, MEAM followed by auto-SCT). Patients who received combination of rituximab and other immunotherapy were excluded as they generally were not indicated for treatment of rrDLBCL.

**Fig 1:**
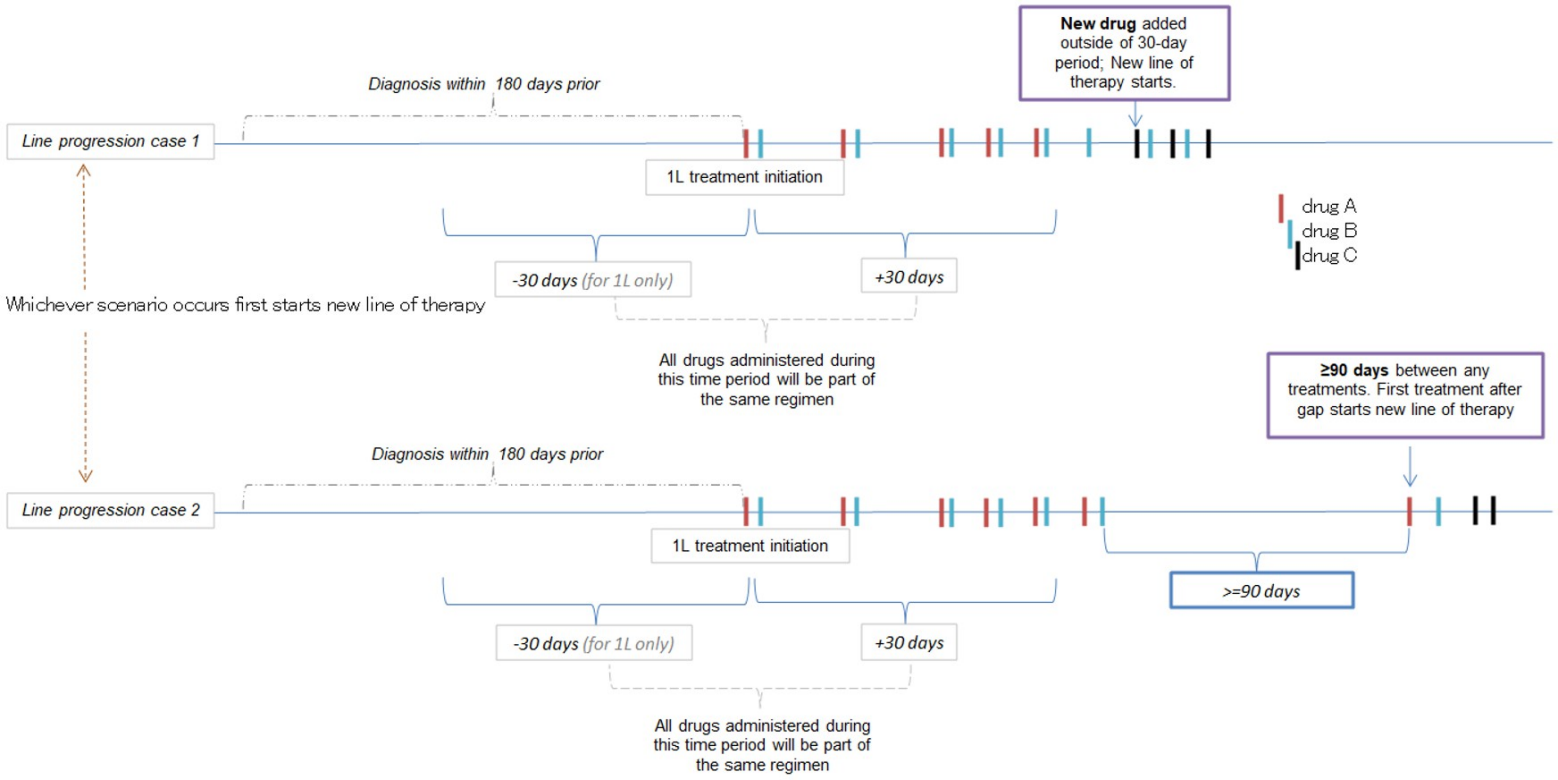
Line of therapy diagram indicating identification of treatment lines.

Patients were considered to be the same line of therapy if they were on the same regimen without a gap. Thus, a treatment regimen was considered a new line of therapy if the patient took a drug not included in their initial treatment regimen more than 30 days after treatment initiation date, or had a gap in treatment for >90 days (Fig 1). Patients who had a record of SCT (allogeneic (allo)- or auto-) were also considered part of the same line of therapy if the transplant occurred prior to a next line of therapy as described above. The approach for defining lines of therapy has also been previously described [16].

### Healthcare resource utilization

Healthcare resources used during follow-up were assessed and included: number of patients receiving each line of therapy (i.e. 2, 3, 4, 5+), hospitalizations, ICU admissions, emergency room visits, any imaging (positron emission tomography (PET) scans, magnetic resonance imaging (MRI), computerized tomography (CT) scans), allogenic SCT (allo-SCT), auto-SCT, and radiation therapy. Mean (SD), median (Q1, Q3) and minimum and maximum values were calculated for continuous data and categorical data was calculated as the number of patients and proportion of the cohort.

### Medical costs

Medical costs were the main outcomes of interest of the SEM. Total medical cost of care was calculated to include both DLBCL-related and DLBCL non-related costs, which occurred during each patient’s follow-up (from the 2L/3L treatment). The components of total costs were: inpatient cost, intensive care unit (ICU) cost, outpatient cost, cancer treatment costs, and other pharmacy costs (for drugs prescribed other than cancer treatment). SCT costs, including any allo-SCT and auto-SCT. In addition to the SEM, all of these cost components were described as the number of patients, mean (SD), median (Q1, Q3), as well as minimum and maximum values.

Nominal direct medical costs were obtained in Japanese yen (JPY) directly from the database. Direct unadjusted (nominal) medical costs were presented after converting from JPY to USD using the exchange rate based on the first month of every year [17]. Direct unadjusted (nominal) medical costs were then adjusted to direct adjusted medical costs with regard to Japanese inflation rate based on the calendar year average of Consumer Price Index (reference year: 2020) [18].

### Statistical analysis

The primary outcome for this study was the drivers of total medical cost. A SEM with path analysis was constructed to assess medical cost drivers as the associations with and between patient profile components (e.g. treatment regimen received, demographics, clinical conditions and HCRU) and total medical cost. The SEM is a measurement model used to define complex relationships between observed variables and their underlying concepts [19]. SEM includes two major components, a measurement model assessing confirmatory factor analysis and structural model for multiple regression/path analysis [20]. As the input parameters were not conceptual and defined from claims data, the model was constructed as path analyses, and due to the skew of medical cost and sample size under 5000, non-normality was accounted for with robust standard error [21, 22].

**Fig 2:**
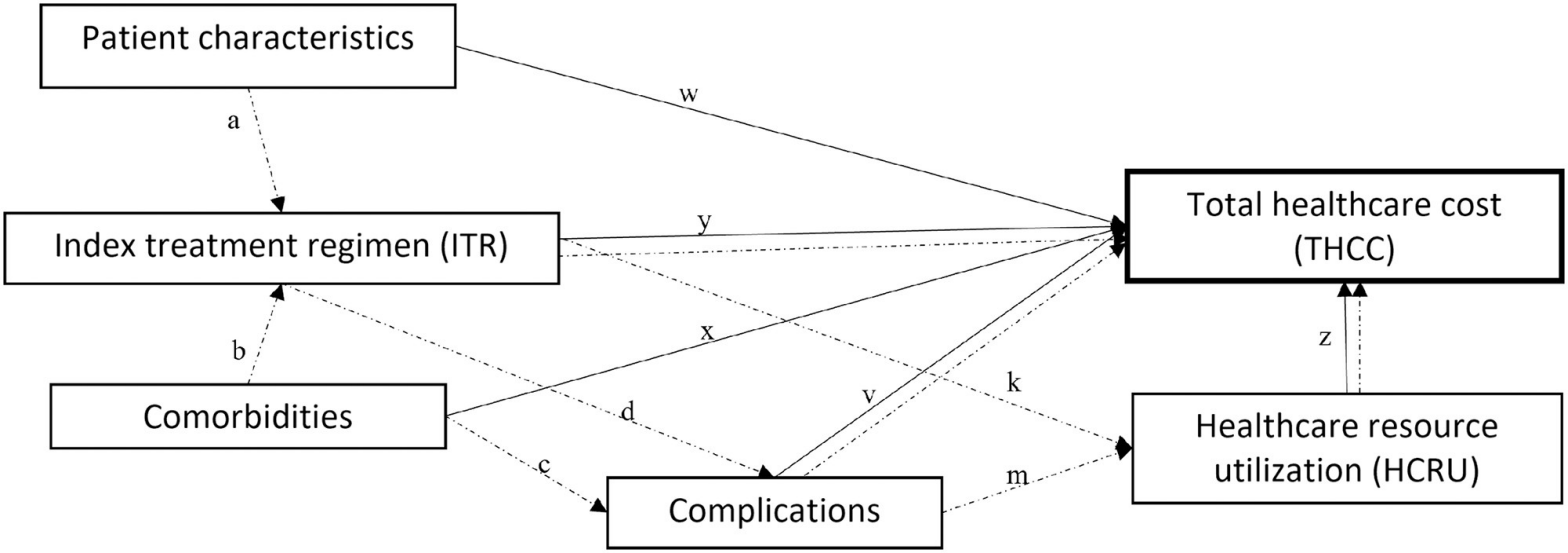
SEM pathway diagram used for each cohort. Solid lines indicate relationship specified as direct effect on THCC in the model and dotted lines indicate relationship specified as indirect effect on THCC via mediator(s) in the model.

All effects observed upon analysis with SEM are presented as direct, indirect and total effects for each cohort. The results of each effect category are presented as coefficients (B), standardize coefficients (β) with 95% confidence intervals (95%CI), and a two-sided test for significance (*p*-value). As the conventional of presentation of parameter estimates are the standardized coefficients its level of significance (p<0.05 or p<0.01) [23, 24], threshold for all SEM coefficients was therefore set at 5%. The goodness of fit was tested for both SEMs using the standardized root mean square residual (SRMR), in which a value less than 0.08 is considered a well-fitted model [25]. The SEM pathway diagram showing the hypothesized relationships between variables is presented in Fig 2. Based on prior literature on covariates related to medical cost found in literature, patient clinical characteristics, treatment regimen, comorbidities, and complications were theorized to be direct effects on total healthcare cost in the SEM. Given the nature of the retrospective database, as medical cost is directly derived from an associated procedure or treatment, HCRU was also specified as a direct effect. Index treatment regimen was additionally specified as a mediator, as patient characteristics and comorbidities may also affect treatment regimen, and thus indirectly the medical cost. Similarly, complications and HCRU were also specified as mediators, as comorbidities and index regimen may indirectly impact total medical cost due to certain complications and high HCRU. Total effect for each predictor was subsequently calculated as the sum of the direct and indirect effects.

#### Direct effects

- Total healthcare cost (THCC)~ w * Patient Characteristics + x * Comorbidities + y * index treatment regimen (ITR) + v * Complications + z * HCRU

#### Mediators

- ITR ~ a * PatientCharacteristics + b * Comorbidities
- HCRU ~ k * ITR + m * complications
- Complications ~ c * Comorbidities + d * ITR

#### Indirect effects

- Patient characteristics (indirect): = a * y + a * k * z + a * d * v + a * d * m * z
- Comorbidities (indirect): = b * y +b * k * z + b * d * m * z + c * v + c * m * z
- ITR (indirect): = k * z + d * m * z + d * v
- Complications (indirect): = m * z

#### Total effects

- Total,Patient characteristics: = w + patient characterisics (indirect)
- Total, Comorbidities: = x + comorbidities (indirect)
- Total, ITR: = y +ITR (indirect)
- Total, Complications: = v +complications (indirect)
- Total, HCRU: = z

The DLBCL analytical dataset was obtained from SAS^®^ version 9.4 or higher, and all SEM data analyses were performed using the Lavaan package in R [26].

## Results

**Fig 3:**
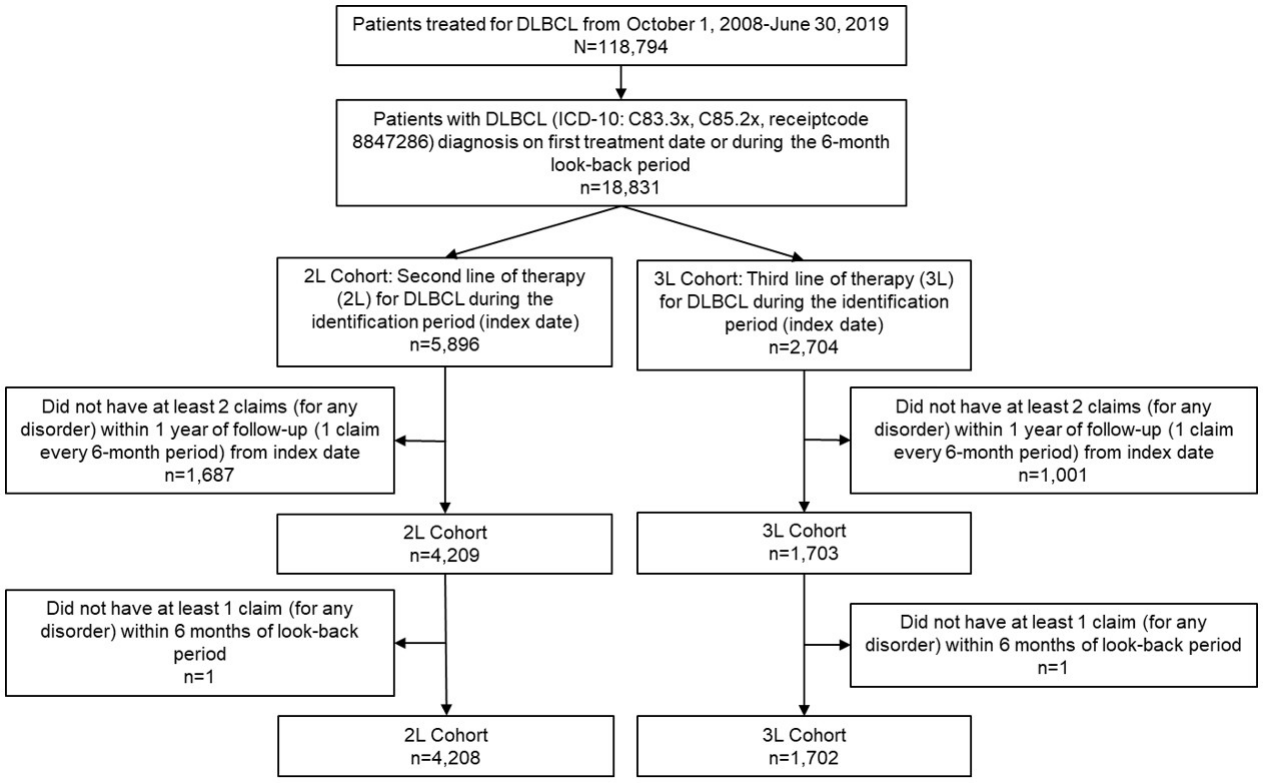
Selection flow for patients with rrDLBCL in each cohort.

There were 4,208 patient records included in the 2L cohort and 1,702 patient records in the 3L cohort (Fig 3).

### Patient profile of 2L cohort

Patient profiles for both cohorts are presented for several key characteristics in Table 1. In the 2L cohort 55.7% were male and the mean [median] (SD) age was 68.9 [70.0] (12.4) years. The largest age group proportion was those under 66 years old (32.9%). The index year of treatment for the 46.9% of the 2L patients was on or after 2017. Mean [median] (SD) follow-up time was 916.2 [685.0] (694.1) days.

**Table 1:** Characteristics of rrDLBCL patient cohorts.

	2L Cohort		3L Cohort	
**Number of patients**, N	4208		1702	
**Gender**, n (%)				
Female	1864	(44.3)	752	(44.2)
Male	2344	(55.7)	950	(55.8)
**Age**				
Mean (SD)	68.9 (12.4)		67.7 (12.4)	
Median (Q1, Q3)	70.0 (62.0, 78.0)		69.0 (61.0, 77.0)	
Min, max	6.0, 97.0		17.0, 95.0	
**Age groups**, n (%)				
<66	1386	(32.9)	628	(36.9)
66–80	2133	(50.7)	848	(49.8)
>80	689	(16.4)	226	(13.3)
**Index year**, n (%)				
2008–2010	96	(2.2)	28	(1.7)
2011–2015	1527	(36.3)	584	(34.3)
2016–2019	2585	(61.4)	1090	(64.1)
**Follow-up time (from index date until death or last patient record)**, n (%)				
Mean (SD)	916.2 (694.1)		820.6 (650.0)	
Median (Q1, Q3)	685.0 (381.0, 1279.0)		581.5 (331.0, 1132.0)	
Min, max	182.0, 4109.0		182.0, 4062.0	
**Complications, n (%)**				
Heart disease*	670	(15.9)	246	(14.5)
Kidney disease*	220	(5.2)	95	(5.6)
Liver disease*	680	(16.2)	252	(14.8)
**Prior SCT**, n (%)	570	(13.5)	394	(23.1)
**Prior radiation therapy**, n (%)	1376	(32.7)	625	(36.7)
**Modified baseline CCI**†, n (%)				
0–2	1945	(46.2)	708	(41.6)
3	479	(11.4)	201	(11.8)
4	609	(14.5)	257	(15.1)
5+	1175	(27.9)	536	(31.5)

CCI, Charlson Comorbidity Index

*New diagnosis after index date, with no history of disease any prior respective lines

^†^Modified by removing DLBCL as a comorbidity

Nearly one third of patients had prior radiation therapy (32.7%), and a small proportion had prior SCT (13.5%). The mean [median] (SD) duration of 2L regimen was 3.7 [2.4] (5.5) months (**S1 Table in** S1 File). There were 20.3% and 22.0% of patients with congestive heart failure and chronic pulmonary disease, respectively. The proportion of the 2L group with baseline CCI score of 5 or greater decreased from 31.0% to 27.9% after removing DLBCL diagnosis from the calculation of the CCI score.

### Patient profile of 3L cohort

In the 3L cohort, 55.8% were male and the mean [median] (SD) age was 67.7 [69.0] (12.4) for the entire population. A minority of patients were aged 71 years or above (45.1%). The index year of treatment for the 49.5% of these patients was on or after 2017. Mean [median] (SD) follow-up time was 820.6 [581.5] (650.0) days. A large minority of patients had prior radiation therapy (36.7%) or prior SCT (23.1%). The mean [median] (SD) duration of 2L in the 3L cohort was 2.6 [1.9] (3.1) months (**S1 Table in** S1 File). Comorbidities were identified in many 3L patient records, including 24.5% and 25.8% of patients with congestive heart failure and chronic pulmonary disease, respectively. Mild liver disease and metastatic solid tumors were also found in 27.4% and 18.2% of patients, respectively. Almost one third of patients had a CCI score of 5 or greater (31.5%) even after removing DLBCL diagnosis from the calculation.

While proportions of patients receiving multiple lines of treatment differed slightly, the most common treatment categories for the 2L and 3L cohorts followed similar patterns (Table 2). In both cohorts, gemcitabine, dexamethasone, cisplatin/carboplatin (GDP)-based with or without rituximab was the most common specific regimen across all treatment lines (range: 7.7%-9.0%). The largest minority of the 2L cohort (44.4%) of patients received other R-based therapy in 2L. This proportion decreased to 21.6% by 5L for the 2L cohort and 22.7% for the 5L of the 3L cohort. During 3L for the 2L cohort, 24.9% received other R-based therapy and 28.1% received other chemotherapy without R. The 3L regimen for 3L cohort, in contrast, was comprised evenly of other R-based (32.7%) therapies or other chemotherapy without rituximab (30.4%). The proportion of patient receiving other R-based regimens switched to other chemotherapy without rituximab by 3L and steadily increased as patients progressed to through 5L. Very few patients received induction regimens prior auto-SCT, detailed in **S2 Table in** S1 File. HCRU for 2L and 3L cohorts was relatively similar with the exception of transplantation outcomes. For example, the mean [median] (SD) number of hospitalizations for the 2L cohort was 5.0 [4.0] (3.8) with a mean [median] (SD) length of hospital stay (LOS) of 122.9 [100.0] (100.5) days. For mean [median] (SD) number of hospitalizations, the 3L cohort had 5.0 [4.0] (3.9) with a mean [median] (SD) LOS of 126.3 [100.5] (103.0) days. ICU admissions and emergency room visits were rare at less than 5% for both cohorts. It was notable that 13.3% of 2L cohort patients received an auto-SCT but 21.4% of 3L cohort patients received the same kind of transplantation during the follow-up period.

**Table 2:** Treatment patterns for rrDLBLC patients.

	2L Cohort		3L Cohort	
**Number of treatment patients**, N	4208		1702	
Total lines, mean (SD)	3.5 (1.7)		4.8 (1.8)	
2, n (%)	1432	(34.0)	-	-
3, n (%)	1096	(26.0)	485	(28.5)
4, n (%)	714	(17.0)	424	(24.9)
5+, n (%)	966	(23.0)	793	(46.6)
**Second line regimen (2L),** n (%)				
R+/-DeVIC-based	250	(5.9)	-	-
R-CHASE-based	280	(6.7)	-	-
GDP-based with or without R	323	(7.7)	-	-
R-Bendamustine-based	98	(2.3)	-	-
R-EPOCH	143	(3.4)	-	-
R-ESHAP-based	70	(1.7)	-	-
ESHAP-based	24	(0.6)	-	-
R-ICE-based	21	(0.5)	-	-
R-DHAP-based	3	(0.1)	-	-
Other R-based	1868	(44.4)	-	-
Before an auto-SCT regimens (MEAN, LEED, MCEC, MEAM)*	52	(1.2)	-	-
Other chemotherapy without R	1076	(25.6)	-	-
**Third line regimen (3L),** n (%)				
R+/-DeVIC-based	186	(6.7)	111	(6.5)
R-CHASE-based	112	(4.0)	74	(4.3)
GDP-based with or without R	234	(8.4)	140	(8.2)
R-Bendamustine-based	71	(2.6)	47	(2.8)
R-EPOCH	114	(4.1)	104	(6.1)
R-ESHAP-based	52	(1.9)	40	(2.4)
ESHAP-based	31	(1.1)	19	(1.1)
R-ICE-based	22	(0.8)	15	(0.9)
R-DHAP-based	0	0.0	0	0.0
Other R-based	691	(24.9)	557	(32.7)
Before an auto-SCT regimens (MEAN, LEED, MCEC, MEAM)*	89	(3.2)	78	(4.6)
Other chemotherapy without R	781	(28.1)	517	(30.4)
Not otherwise specified	393	(14.2)		NA
**Fourth line regimen (4L),** n (%)				
R+/-DeVIC-based	124	(7.4)	87	(7.1)
R-CHASE-based	66	(3.9)	52	(4.3)
GDP-based with or without R	152	(9.0)	108	(8.9)
R-Bendamustine-based	45	(2.7)	34	(2.8)
R-EPOCH	56	(3.3)	43	(3.5)
R-ESHAP-based	29	(1.7)	16	(1.3)
ESHAP-based	10	(0.6)	7	(0.6)
R-ICE-based	10	(0.6)	9	(0.7)
R-DHAP-based	3	(0.2)	3	(0.2)
Other R-based	362	(21.5)	294	(24.2)
Before an auto-SCT regimens (MEAN, LEED, MCEC, MEAM)*	26	(1.5)	24	(2.0)
Other chemotherapy without R	530	(31.5)	386	(31.7)
Not otherwise specified	267	(15.9)	154	(12.7)
**Subsequent regimen (5L),** n (%)				
R+/-DeVIC-based	70	(7.2)	55	(6.9)
R-CHASE-based	24	(2.5)	22	(2.8)
GDP-based with or without R	84	(8.7)	67	(8.4)
R-Bendamustine-based	19	(2.0)	16	(2.0)
R-EPOCH	17	(1.8)	17	(2.1)
R-ESHAP-based	11	(1.1)	10	(1.3)
ESHAP-based	11	(1.1)	10	(1.3)
R-ICE-based	5	(0.5)	4	(0.5)
R-DHAP-based	0	0.0	0	0.0
Other R-based	209	(21.6)	180	(22.7)
Before an auto-SCT regimens (MEAN, LEED, MCEC, MEAM)*	13	(1.3)	10	(1.3)
Other chemotherapy without R	349	(36.1)	293	(36.9)
Not otherwise specified	154	(15.9)	109	(13.7)

R, rituximab; DeVIC, dexamethasone, etoposide, ifosfamide, carboplatin; CHASE, cyclophosphamide, cytarabine, etoposide, dexamethasone; GDP, gemcitabine, dexamethasone, cisplatin/carboplatin; Bendamustine; EPOCH, etoposide, prednisolone, vincristine, cyclophosphamide, doxorubicin; ESHAP, etoposide, cytarabine, cisplatin, methylprednisolone; ICE, ifosfamide, carboplatin, etoposide; DHAP, dexamethasone, cytarabine, cisplatin; MINE, mitoxantrone, ifosfamide, mesna, etoposide; LEED, melphalan, cyclophosphamide, etoposide, dexamethasone; MCEC, ranimustine, carboplatin, etoposide, cyclophosphamide; MEAM, ranimustine, etoposide, cytarabine, melphalan

* Includes only patients who underwent auto-SCT after regimen; patients who underwent the following therapies but did not undergo auto-SCT after the regimen were counted as "Other chemotherapy without R"

Medical costs for the cohorts were comparable (Table 3), with the 3L cohort having slightly higher total follow-up costs compared to 2L (by less than 2,000 USD). The mean [median] (SD) total medical cost of care for the 2L cohort was 73,296.40 [58,223.11] (58,409.79) USD. Inpatient costs were the highest component of total cost (mean [median]; 60,941.71 [47,026.10] USD). The mean [median] (SD) total medical cost of care for the 3L cohort was 75,238.35 [60,477.31] (59,583.66) USD where inpatient costs were also the highest component of total cost (mean [median]; 64,081.09 [49,795.46] USD). Total costs as well as relative follow-up time stratified by age and gender are shown in **S3 Table in** S1 File. While older age correlated less follow-up time and subsequently lower unadjusted total cost, female patients had comparable follow-up time with male patients, yet accrued lower total cost.

**Table 3:** Healthcare costs (USD) during follow-up.

	2L Cohort	3L Cohort
**Total medical cost of care**, N	4208	1702
Mean (SD)	73296.40 (58409.79)	75238.35 (59583.66)
Median (Q1, Q3)	58223.11 (32898.90, 94623.89)	60477.31 (33081.40, 98382.96)
Min, Max	1842.95, 528090.15	1893.19, 449778.98
**Cost subcategories**		
**Inpatient cost**, n	3965	1596
Mean (SD)	60941.71 (53470.41)	64081.09 (54397.09)
Median (Q1, Q3)	47026.10 (22754.39, 82425.81)	49795.46 (25324.70, 87557.80)
Min, Max	488.50, 415774.22	466.74, 382401.46
**Intensive care unit (ICU) cost**	112	42
Mean (SD)	4282.24 (4184.19)	3553.31 (3656.36)
Median (Q1, Q3)	2510.72 (866.34, 6485.93)	2015.99 (938.02, 5198.07)
Min, Max	791.53, 16611.09	791.53, 16611.09
**Outpatient cost**	4101	1661
Mean (SD)	16288.06 (22139.76)	15522.13 (22552.28)
Median (Q1, Q3)	9806.97 (4434.62, 20371.38)	8807.34 (3904.47, 18604.34)
Min, Max	6.76, 364765.41	8.64, 361559.02
**Cancer treatment costs**	4208	1702
Mean (SD)	16027.24 (19331.29)	14979.17 (20362.97)
Median (Q1, Q3)	11909.82 (5896.15, 20001.11)	9921.29 (4487.80, 18916.99)
Min, Max	6.43, 398654.49	10.17, 365816.51
**Other pharmacy costs**	4206	1702
Mean (SD)	17709.69 (23990.57)	19601.32 (24941.91)
Median (Q1, Q3)	9985.52 (4407.57, 21099.57)	11206.48 (4713.06, 23497.90)
Min, Max	-9874.54, 309876.04	8.99, 254909.73
**Any SCT costs**	566	366
Mean (SD)	3214.07 (1084.62)	3161.93 (1100.12)
Median (Q1, Q3)	2840.14 (2758.10, 3091.32)	2840.14 (2666.34, 3091.32)
Min, Max	2234.19, 12090.71	2234.19, 12090.71
**Allo-SCT costs**	32	12
Mean (SD)	6235.67 (1130.49)	6690.51 (1773.38)
Median (Q1, Q3)	6117.58 (5842.05, 6149.83)	6149.83 (6029.23, 6149.83)
Min, Max	5618.75, 12090.71	5618.75, 12090.71
**Auto-SCT costs**	558	364
Mean (SD)	3164.70 (1014.60)	3130.08 (1041.24)
Median (Q1, Q3)	2840.14 (2758.10, 3091.32)	2840.14 (2666.34, 3091.32)
Min, Max	2234.19, 12090.71	2234.19, 12090.71

*Direct unadjusted (nominal) medical costs are presented after converting from JPY to USD using the exchange rate based on the first month of every year.

### SEM outcomes

Estimates of total effects on medical cost, and its component indirect and direct effects, are presented for the 2L and 3L cohorts (Table 4).

**Table 4:** Standardized estimates from structural equation model of 2L and 3L cost drivers.

THCC drivers	Direct effect (USD)				Indirect effect^#^ (USD)				Total effect (USD)			
	β	95%CI		*p*	β	95%CI		*p*	β	95%CI		*p*
***2L***												
**Patient characteristics**												
Gender (reference: male)												
Female	-0.036	-0.052	-0.020	<0.001	0	-0.007	0.007	0.927	**-0.036**	**-0.053**	**-0.019**	<0.001
Age (reference: <66)												
66–70	-0.030	-0.047	-0.013	0.001	-0.009	-0.017	0	0.043	**-0.038**	**-0.057**	**-0.020**	<0.001
71–75	-0.052	-0.068	-0.036	<0.001	-0.013	-0.023	-0.004	0.004	**-0.065**	**-0.083**	**-0.048**	<0.001
76–80	-0.063	-0.078	-0.047	<0.001	-0.016	-0.025	-0.007	0.001	**-0.079**	**-0.096**	**-0.061**	<0.001
81–85	-0.081	-0.096	-0.066	<0.001	-0.02	-0.028	-0.011	<0.001	**-0.101**	**-0.116**	**-0.085**	<0.001
85+	-0.063	-0.075	-0.052	<0.001	-0.023	-0.030	-0.017	<0.001	**-0.086**	**-0.099**	**-0.073**	<0.001
Index year	-0.119	-0.136	-0.102	0	0.006	-0.002	0.014	0.128	**-0.113**	**-0.131**	**-0.094**	<0.001
**Comorbidities**												
CCI score (reference: 0–2)												
3	0.017	0	0.034	0.051	-0.009	-0.021	0.003	0.127	0.008	-0.013	0.029	0.454
4	0.018	0.001	0.034	0.035	-0.040	-0.051	-0.028	<0.001	**-0.022**	**-0.042**	**-0.002**	0.028
5+	0.062	0.044	0.081	<0.001	-0.059	-0.073	-0.045	<0.001	0.003	-0.019	0.026	0.768
Prior/concurrent non-lymphoma neoplasms (reference: No)	0.024	0.006	0.041	0.009	-0.003	-0.014	0.008	0.554	0.020	0	0.041	0.054
**Complications**												
Heart disease (reference: No)	0.064	0.042	0.085	<0.001	0.155	0.128	0.181	<0.001	**0.218**	**0.184**	**0.252**	<0.001
Liver disease	0.012	-0.007	0.03	0.210	0.108	0.081	0.134	<0.001	**0.119**	**0.088**	**0.150**	<0.001
Kidney disease	0.053	0.029	0.078	<0.001	0.062	0.033	0.090	0.007	**0.115**	**0.074**	**0.156**	<0.001
**Index treatment regimen** *												
R+/-DeVIC-based	-0.002	-0.017	0.013	0.796	0.110	0.083	0.136	<0.001	**0.108**	**0.078**	**0.137**	<0.001
R-CHASE-based	0.016	-0.005	0.037	0.136	0.175	0.150	0.20	<0.001	**0.191**	**0.157**	**0.225**	<0.001
GDP-based without or without R	-0.006	-0.019	0.008	0.404	0.07	0.043	0.097	<0.001	**0.064**	**0.038**	**0.091**	<0.001
R- Bendamustine -based	0.076	0.050	0.102	0	0.014	-0.015	0.044	0.340	**0.091**	**0.054**	**0.127**	<0.001
R-EPOCH	-0.005	-0.020	0.011	0.556	0.080	0.054	0.107	<0.001	**0.076**	**0.044**	**0.108**	<0.001
R-ESHAP-based	0.004	-0.012	0.019	0.640	0.068	0.043	0.092	<0.001	**0.071**	**0.040**	**0.102**	<0.001
ESHAP-based	-0.006	-0.025	0.012	0.505	0.046	0.021	0.072	<0.001	**0.04**	**0.002**	**0.078**	0.040
R-ICE-based	0.001	-0.009	0.010	0.911	0.059	0.023	0.095	0.001	**0.059**	**0.020**	**0.098**	0.003
R-DHAP-based	0.002	-0.009	0.013	0.720	0.009	0.003	0.015	0.002	0.011	-0.002	0.025	0.091
Other R-based	0.033	0.017	0.049	<0.001	0.002	-0.024	0.028	0.871	**0.035**	**0.005**	**0.065**	0.022
Induction therapy before auto-SCT regimens†	-0.026	-0.044	-0.007	0.007	0.023	0	0.045	0.046	-0.003	-0.031	0.025	0.826
**HCRU**								-				
Number of hospitalizations (reference: No)	0.088	0.064	0.112	<0.001	-	-	-	-	**0.088**	**0.064**	**0.112**	<0.001
Any ICU admission	0.050	0.027	0.072	<0.001	-	-	-	-	**0.050**	**0.027**	**0.072**	<0.001
Any PET scans	0.022	0.005	0.040	0.013	-	-	-	-	**0.022**	**0.005**	**0.040**	0.013
Any MRI scans	0.029	0.013	0.046	0.001	-	-	-	-	**0.029**	**0.013**	**0.046**	0.001
Any CT scans	0.005	-0.008	0.017	0.449	-	-	-	-	0.005	-0.008	0.017	0.449
Any emergency room visits	0.002	-0.012	0.017	0.760	-	-	-	-	0.002	-0.012	0.017	0.760
Any SCT	0.156	0.133	0.178	<0.001	-	-	-	-	**0.156**	**0.133**	**0.178**	0
Any radiation therapy	0.005	-0.011	0.021	0.572	-	-	-	-	0.005	-0.011	0.021	0.572
LOS	0.750	0.728	0.772	<0.001	-	-	-	-	**0.750**	**0.728**	**0.772**	<0.001
Standardized Root Mean Square Residual (SRMR)‡: 0.006												
***3L***												
**Patient characteristics**												
Gender (reference: male)												
Female	-0.032	-0.057	-0.006	0.015	-0.002	-0.012	0.008	0.687	**-0.034**	**-0.061**	**-0.007**	0.014
Age (reference: <66)												
66–70	-0.027	-0.054	0.001	0.057	0.003	-0.009	0.015	0.608	-0.023	-0.053	0.006	0.119
71–75	-0.048	-0.077	-0.018	0.001	0.003	-0.010	0.016	0.648	**-0.045**	**-0.075**	**-0.014**	0.004
76–80	-0.076	-0.100	-0.052	<0.001	0.003	-0.011	0.017	0.664	**-0.073**	**-0.099**	**-0.048**	<0.001
81–85	-0.054	-0.079	-0.03	<0.001	-0.001	-0.013	0.012	0.919	**-0.055**	**-0.082**	**-0.028**	<0.001
85+	-0.063	-0.087	-0.039	<0.001	-0.009	-0.018	-0.001	0.037	**-0.072**	**-0.096**	**-0.049**	<0.001
Index year	-0.116	-0.141	-0.091	<0.001	-0.001	-0.013	0.010	0.823	**-0.117**	**-0.144**	**-0.090**	<0.001
**Comorbidities**												
CCI score (reference: 0–2)												
3	0.008	-0.022	0.037	0.612	-0.013	-0.028	0.003	0.103	-0.005	-0.038	0.028	0.772
4	0.017	-0.009	0.042	0.203	-0.036	-0.053	-0.018	<0.001	-0.019	-0.049	0.011	0.213
5+	0.056	0.025	0.088	<0.001	-0.055	-0.077	-0.033	<0.001	0.001	-0.035	0.037	0.950
Prior/concurrent non-lymphoma neoplasms (reference: No)	0.037	0.008	0.066	0.012	-0.019	-0.034	-0.004	0.013	0.018	-0.015	0.051	0.281
**Complications**												
Heart Disease (reference: No)	0.058	0.027	0.089	<0.001	0.118	0.075	0.161	<0.001	**0.176**	**0.122**	**0.230**	<0.001
Liver Disease	0.021	-0.009	0.051	0.167	0.098	0.054	0.141	<0.001	**0.119**	**0.067**	**0.171**	<0.001
Kidney Disease	0.046	0.011	0.08	0.009	0.063	0.017	0.108	0.007	**0.108**	**0.049**	**0.168**	<0.001
**Index treatment regimen** *												
R+/-DeVIC-based	0	-0.022	0.022	0.991	0.082	0.041	0.122	0	**0.082**	**0.034**	**0.129**	0.001
R-CHASE-based	0.008	-0.019	0.035	0.546	0.107	0.065	0.15	0	**0.116**	**0.059**	**0.172**	<0.001
GDP-based without or without R	-0.007	-0.031	0.018	0.603	0.043	0.001	0.086	0.046	0.037	-0.004	0.077	0.076
R- Bendamustine -based	0.058	0.023	0.094	0.001	-0.026	-0.063	0.012	0.184	0.033	-0.014	0.079	0.169
R-EPOCH	-0.020	-0.040	0	0.048	0.018	-0.018	0.054	0.323	-0.002	-0.043	0.039	0.918
R-ESHAP-based	-0.001	-0.020	0.018	0.892	0.043	0.018	0.069	0.001	**0.042**	**0.009**	**0.074**	0.011
ESHAP-based	0.001	-0.027	0.030	0.932	0.015	-0.012	0.042	0.262	0.017	-0.029	0.063	0.475
R-ICE-based	0.046	0.008	0.084	0.018	0.079	0.020	0.137	0.008	**0.125**	**0.050**	**0.200**	0.001
Other R-based	0.047	0.020	0.074	0.001	-0.049	-0.091	-0.007	0.024	-0.002	-0.052	0.048	0.939
Induction therapy before auto-SCT regimens†	-0.023	-0.051	0.005	0.106	0.015	-0.019	0.049	0.381	-0.008	-0.053	0.037	0.727
**HCRU**												
Number of hospitalizations (reference: No)	0.121	0.085	0.157	<0.001	-	-	-	-	**0.121**	**0.085**	**0.157**	<0.001
Any ICU admission	0.049	0.012	0.087	0.010	-	-	-	-	**0.049**	**0.012**	**0.087**	0.0100
Any PET scans	0.023	-0.004	0.051	0.100	-	-	-	-	0.023	-0.004	0.051	0.100
Any MRI scans	0.025	-0.001	0.052	0.062	-	-	-	-	0.025	-0.001	0.052	0.062
Any CT scans	0.014	-0.003	0.031	0.104	-	-	-	-	0.014	-0.003	0.031	0.104
Any emergency room visits	0.009	-0.017	0.036	0.490	-	-	-	-	0.009	-0.017	0.036	0.490
Any SCT	0.154	0.120	0.187	<0.001	-	-	-	-	**0.154**	**0.120**	**0.187**	<0.001
Any radiation therapy	0.005	-0.021	0.030	0.722	-	-	-	-	0.005	-0.021	0.030	0.722
LOS	0.762	0.729	0.794	<0.001	-	-	-	-	**0.762**	**0.729**	**0.794**	<0.001
Standardized Root Mean Square Residual (SRMR)‡: 0.065												

β, standardized coefficient; 95%CI, 95% confidence interval; ICU, intensive care unit; ITR, index treatment regimen; LOS, length of hospital stay; THCC, total health care cost; HCRU, healthcare resource utilization; USD, US dollars.

^#^Represents the total indirect effect of variable on THCC via all specified mediators. Paths and mediators for the variables under each category are described in Fig 2 and methods section

*Reference treatment group = other chemotherapy without rituximab

^†^Includes only patients who underwent auto-SCT after regimen; patients who underwent induction therapies but did not undergo auto-SCT after the regimen were counted as "Other chemotherapy without R"

^‡^Hu and Bentler,1999: SRMR of <0.08 represents a well-fitted model

LOS had the largest total effect on medical cost in the 2L cohort (β: 0.750 [95%CI: 0.728, 0.772]). The other largest cost drivers were heart disease complications (β: 0.218, [95%CI: 0.184, 0.252]), having R-CHASE treatment regimen as an index regimen (β: 0.191 [95%CI: 0.157, 0.225]) and induction regimens with auto-SCT (β: 0.156, [95%CI: 0.133, 0.178]). The largest protective drivers were all older age groups compared to those under 66 years, increasing index year (β: -0.113 [95%CI: -0.131, -0.094]), female gender (β: -0.036 [95%CI: -0.053, -0.019]), and a CCI score of 4 (β: -0.022 [95%CI: -0.042, -0.002]). The 81–85 age group had the strongest negative relationship with medical cost (β: -0.101 [95%CI: -0.116, -0.085]). This model fit the data well with a SRMR of 0.060.

The total effect of LOS on medical cost was also the largest for the 3L cohort (β: 0.762 [95%CI: 0.729, 0.794]). The next largest driver of total cost was having heart disease as a complication (β: 0.176 [95%CI: 0.122, 0.230]), with a large part due to the indirect effects (β: 0.118 [95%CI: 0.075, 0.161]) and any SCT (β: 0.154 [95%CI: 0.120, 0.187]). Unlike the 2L cohort where it was third largest, R-CHASE as an index regimen was associated with the fourth largest increase in cost burden in the 3L cohort (β: 0.116 [95%CI: 0.059, 0.172]). Parameters associated with significant decrease in burden of cost included female gender (β: -0.034 [95%CI: -0.061, -0.007]), older age groups (71 years or older) compared to those under 66 years, and increasing index year of treatment (β: -0.117 [95%CI: -0.144, -0.090]). CCI score was not significantly associated with total effect on medical cost. This model fit the data well with a SRMR of 0.065. Other prior or concurrent primary cancers besides DLBCL did not have a total effect on cost for either 2L or 3L cohort.

## Discussion

Total medical cost during follow-up was relatively similar between 2L and 3L cohorts with average costs for 2L of 73,296 USD and 75,238 USD for 3L patients. The two treatment cohorts of rrDLBCL patients had similar baseline characteristics, HCRU, cost and cost drivers, except a few notable exceptions in terms of relative cost driver size. LOS and heart disease complications were consistently the largest drivers of medical costs was for both 2L and 3L cohorts. In 3L, the effect of LOS was about four times larger than heart disease complications and LOS was about three times larger than the effect of heart disease complications in 2L. The 2L cohort had about one third fewer auto-SCT than the 3L cohort and SCT was the third largest cost driver in the 3L cohort compared to R-CHASE regimen in the 2L cohort. These differences may reflect complex clinical decision-making about curative treatments based on the baseline characteristics of patients who have rrDLBCL refractory to more than one line of salvage chemotherapy in Japan.

The biggest cost driver was LOS followed by heart disease complications for both cohorts. In 3L, the effect of LOS was about four times largest than heart disease complications and in 2L LOS was about three times larger than the effect of heart disease complications. This large effect of LOS is distinct from other studies using SEM to calculate effects on medical cost. For example, an SEM path analysis of respiratory syncytial virus in Japanese children found the effect of LOS on medical cost to be high but approximately 10 times lower than the effect of blood transfusions [27]. In the present study there were also SEM parameters for cohorts where direct effects were positive and the indirect effects were negative or vice versa. Indirect effects tended to be much larger than direct effects thus contributed more to the total effects thus underscoring the importance of realistic model design based on past literature to capture all pertinent effects.

The intersection of cohort patient characteristics and their treatment patterns are one suggestion that there are differences in how more advanced rrDLBCL patients are treated in Japanese real-world practice. The 2L cohort was only slightly older in age than 3L cohort, however a large proportion of 3L patients had more comorbidities than 2L. SCT is considered to be the optimal treatment option for eligible patients with rrDLBCL [28], but within the follow-up period of the current analysis, the 2L cohort had about one third smaller proportion of auto-SCT than the 3L cohort. This may potentially be due to patients receiving their high-dose chemotherapy (HDC) more than 30 days after second line initiation or with a 90-day treatment gap, thus were counted as third line treatment and resulting in slightly higher proportion of SCTs counted in the 3L cohort. Due to the complexity of claims data and the high heterogeneity of salvage regimen drugs, HDC drugs, as well as timing of HDC, explicit separation between salvage chemotherapy and HDC was not further conducted. On the other hand, in spite of their comorbidities, the poorer prognosis of the 3L cohort may have required intensive therapy as conditioning for auto-SCT to further prolong survival. For example, a study of rrDLBCL patients in a single center in the UK found a considerable drop in complete response rates for rrDLBCL with 2L (27.0%), to 3L (17.5%), to 4L (2.4%) [29]. HCST also had one of largest effects on medical cost for both cohorts, though it was relatively higher in 3L. A study of Canadian patients similarly found that SCT had a larger impact on medical cost for patient’s receiving more than one treatment DLBCL [30].

There were several protective factors for medical costs. Increasing age was associated with decreases in cost, mostly due to the shorter survival time (thus observation period) of older patients. Similarly, patients with later index years had a shorter observation period, thus index year was adjusted for in the model, but its coefficient should be interpreted with caution. Exploratory analysis of medical costs for each age group shows decreasing cost with age outside of the SEM, as well as decreasing follow-up time with age. The total effects from SEM results showed that females had significantly less cost burden. Outside of the SEM, females also had lower costs with comparable follow-up time.

The real world treatment patterns used to treat rrDLBCL in Japan are diverse and have different impact on overall medical cost. This treatment has been shown to have some efficacy in rrDLBCL in a phase II study (overall response rate 67%) [31] but this treatment has not been studied in detail from an economic perspective [28].

This study poses a few limitations. First, due to the nature of retrospective claims studies, patients cannot be traced longitudinally and each exact line of therapy assigned may be subject to bias. Additionally, medical costs accrued outside of the facilities captured by the database are not accounted for, which may contribute to an underestimation of the total medical costs. Lastly, due to the complex paths used and the large number of predictors, statistical significance should be interpreted with caution and should be interpreted holistically.

This study is the first in Japan to investigate the relationship between patient attributes, healthcare utilization, and total medical cost in rrDLBCL patients. Our study positioned a holistic model of the predictors of medical drivers in a complex disease with poor prognosis. The findings suggest that although age and gender have direct impact on total cost in both 2L and 3L, complications and treatment regimen also impact total cost, largely through indirect effects.

## Supporting information

S1 File (DOCX)
