# Supplementary material for: Cost drivers associated with diffuse large B-cell lymphoma (DLBCL) in Japan: A structural equation model (SEM) analysis
Source: PLoS One. 2022 May 27;17(5):e0269169. doi: 10.1371/journal.pone.0269169 (PMC9140275; doi:10.1371/journal.pone.0269169)
Supplement: S1 File — (DOCX) [file pone.0269169.s001.docx]

Table S1. Other patient characteristics

|  | **2L Cohort** | | **3L Cohort** | |
| --- | --- | --- | --- | --- |
| **Baseline CCI** | **n** | **(%)** | **n** | **(%)** |
| 0-2 | 1564 | (37.2) | 537 | (31.6) |
| 3 | 529 | (12.6) | 208 | (12.2) |
| 4 | 810 | (19.2) | 352 | (20.7) |
| 5+ | 1305 | (31.0) | 605 | (35.5) |
| **Charlson comorbidities** |  |  |  |  |
| Congestive heart failure | 855 | (20.3) | 417 | (24.5) |
| Dementia | 68 | (1.6) | 28 | (1.7) |
| Chronic pulmonary disease | 925 | (22.0) | 439 | (25.8) |
| Rheumatologic disease | 188 | (4.5) | 66 | (3.9) |
| Mild liver disease | 1034 | (24.6) | 467 | (27.4) |
| Diabetes with chronic complications | 151 | (3.6) | 58 | (3.4) |
| Hemiplegia/paraplegia | 69 | (1.6) | 24 | (1.4) |
| Renal disease | 199 | (4.7) | 84 | (4.9) |
| Any malignancy | 4200 | (99.8) | 1695 | (99.6) |
| Malignancies except for DLBCL (index disease) | 3066 | (72.9) | 1247 | (73.3) |
| Moderate/severe liver disease | 35 | (0.8) | 14 | (0.8) |
| Metastatic solid tumor | 655 | (15.6) | 309 | (18.2) |
| HIV | 8 | (0.2) | 4 | (0.2) |
| **Duration of 1L regimen (months)** |  |  |  |  |
| Mean (SD) | 3.3 (2.9) |  | 3.34 (2.7) |  |
| Median (Q1, Q3) | 2.8 (1.4, 4.6) |  | 2.93 (1.5, 4.8) |  |
| Min, max | 0.03, 51.6 |  | 0.03, 33.1 |  |
| **Duration of 2L regimen (months)** |  |  |  |  |
| Mean (SD) | 3.7 (5.5) |  | 2.6 (3.1) |  |
| Median (Q1, Q3) | 2.4 (1.0, 4.3) |  | 1.9 (1.0, 3.5) |  |
| Min, max | 0.03, 82.9 |  | 0.03, 45.2 |  |
| **Duration of 3L regimen (months)*** |  |  |  |  |
| Mean (SD) | 2.9 (5.1) |  | 3.0 (4.8) |  |
| Median (Q1, Q3) | 1.6 (0.6, 3.3) |  | 1.8 (0.6, 3.5) |  |
| Min, max | 0.03, 71.1 |  | 0.03, 69.8 |  |
| *Duration of 3L was available for 2776 (65.9%) out of the original 2L cohort | | | | |

Table S2. Detailed induction regimens before autologous SCT

|  | **2L Cohort** | | **3L Cohort** | |
| --- | --- | --- | --- | --- |
|  | **n** | **(%)** | **n** | **(%)** |
| **Second line regimen (2L)** |  |  |  |  |
| Before an autologous SCT regimens* |  |  |  |  |
| MINE* | 0 | (0.0) |  |  |
| LEED* | 14 | (0.3) |  |  |
| MCEC* | 7 | (0.2) |  |  |
| MEAM* | 31 | (0.7) |  |  |
| **Third line regimen (3L)** |  |  |  |  |
| Before an autologous SCT regimens* |  |  |  |  |
| MINE* | 0 | (0.0) | 0 | (0.0) |
| LEED* | 30 | (1.1) | 24 | (1.4) |
| MCEC* | 15 | (0.5) | 12 | (0.7) |
| MEAM* | 44 | (1.6) | 42 | (2.5) |
| **Fourth line regimen (4L)** |  |  |  |  |
| Before an autologous SCT regimens* |  |  |  |  |
| MINE* | 0 | (0.0) | 0 | (0.0) |
| LEED* | 16 | (1.0) | 15 | (1.2) |
| MCEC* | 2 | (0.1) | 2 | (0.2) |
| MEAM* | 8 | (0.5) | 7 | (0.6) |
| **Subsequent regimen (5L)** |  |  |  |  |
| Before an autologous SCT regimens* |  |  |  |  |
| MINE* | 0 | (0.0) | 0 | (0.0) |
| LEED* | 2 | (0.2) | 2 | (0.3) |
| MCEC* | 4 | (0.4) | 4 | (0.5) |
| MEAM* | 7 | (0.7) | 4 | (0.5) |
| MINE, mitoxantrone, ifosfamide, mesna, etoposide; LEED, melphalan, cyclophosphamide, etoposide, dexamethasone; MCEC, ranimustine, carboplatin, etoposide, cyclophosphamide; MEAM, ranimustine, etoposide, cytarabine, melphalan. * Includes only patients who underwent autologous SCT after regimen; patients who underwent the following therapies but do not undergo autologous SCT after the regimen were counted as "Other chemotherapy without R" | | | | |
|  |  |  |  |  |
|  |  |  |  |  |
|  |  |  |  |  |
|  |  |  |  |  |
|  |  |  |  |  |
|  |  |  |  |  |

Table S3. Comparison of cost and follow-up time by age and gender

|  | Age <66 | Age 66-70 | Age 71-75 | Age 76-80 | Age 81-85 | Age 85+ | Male | Female |
| --- | --- | --- | --- | --- | --- | --- | --- | --- |
| **2L Cohort** |  |  |  |  |  |  |  |  |
| Follow up-time in days, N | 1386 | 742 | 709 | 682 | 482 | 207 | 2344 | 1864 |
| Mean (SD) | 1082.9 (788.6) | 940.8 (673.6) | 898.0 (664.6) | 794.7 (600.8) | 732.7 (544.1) | 600.7 (451.6) | 908.4 (690.6) | 925.9 (698.5) |
| Median (Q1, Q3) | 858.5 (441.0, 1597.0) | 735.5 (411.0, 1317.0) | 692.0 (385.0, 1231.0) | 570.5 (354.0, 1076.0) | 550.0 (335.0, 965.0) | 466.0 (289.0, 736.0) | 679.0 (377.0, 1248.0) | 691.0 (386.0, 1304.0) |
| Total Cost |  |  |  |  |  |  |  |  |
| Mean (SD) | 88215.56 (70792.60) | 75502.49 (55034.06) | 70921.54 (50326.99) | 63518.74 (46679.65) | 56744.61 (45115.20) | 44384.31 (33060.05) | 75839.91 (60766.53) | 70097.90 (55153.66) |
| Median (Q1, Q3) | 69156.41 (37790.43, 115977.03) | 62830.12 (35433.56, 100012.92) | 59554.61 (34146.15, 96134.86) | 53035.65 (32348.73, 79820.80) | 47270.79 (28201.93, 69849.20) | 32687.07 (20025.19, 64473.90) | 60444.68 (34228.29, 95195.76) | 55475.10 (31063.74, 93112.39) |
| **3L Cohort** |  |  |  |  |  |  |  |  |
| Follow up-time in days, N |  |  |  |  |  |  |  |  |
| Mean (SD) | 1010.3 (748.4) | 792.4 (572.9) | 713.5 (558.8) | 660.2 (552.9) | 687.7 (551.8) | 552.9 (414.0) | 805.9 (648.0) | 839.2 (652.5) |
| Median (Q1, Q3) | 777.0 (393.5, 1460.0) | 598.0 (365.0, 1086.0) | 504.5 (305.0, 914.0) | 439.0 (288.0, 820.0) | 471.0 (302.0, 895.0) | 414.5 (242.0, 764.0) | 580.5 (321.0, 1078.0) | 588.5 (339.5, 1190.0) |
| Total Cost |  |  |  |  |  |  |  |  |
| Mean (SD) | 88194.51 (69992.14) | 77099.01 (58072.94) | 72955.20 (50167.59) | 61352.40 (46949.15) | 59096.88 (46897.45) | 47760.76 (29567.64) | 77191.92 (62155.53) | 72770.42 (56110.32) |
| Median (Q1, Q3) | 67698.56 (36455.72, 118168.37) | 60832.36 (36257.36, 105864.80) | 64410.60 (38005.62, 98819.67) | 52015.29 (27817.67, 82865.87) | 47965.02 (27509.91, 77189.21) | 39380.01 (27800.49, 63747.42) | 60647.17 (33037.27, 99501.70) | 59777.26 (33091.08, 95635.30) |

Table S4. Sensitivity analysis of structural equation models after removing 2019 index year patients

|  | Direct effect (USD) | | | | | Indirect effect (USD) | | | | | Total effect | | | | |
| --- | --- | --- | --- | --- | --- | --- | --- | --- | --- | --- | --- | --- | --- | --- | --- |
|  | →THCC | | | | | →ITR→THCC | | | | | →THCC + (→ITR→THCC) | | | | |
|  | B | β | 95%CI | | *p* | B | β | 95%CI | | *p* | B | β | 95%CI | | *p* |
| **2L Cohort** | -0.307 | -0.118 | -0.135 | -0.101 | <0.001 | 0.014 | 0.005 | -0.003 | 0.014 | 0.210 | -0.293 | -0.113 | -0.132 | -0.094 | <0.001 |
| **3L Cohort** | -0.301 | -0.111 | -0.138 | -0.084 | <0.001 | 0.002 | 0.001 | -0.011 | 0.013 | 0.892 | -0.299 | -0.110 | -0.140 | -0.081 | <0.001 |

Table S5. Structural equation model of 2L cost drivers including unstandardized values

|  | Direct effect (USD) | Indirect effect (USD) | Total effect |
| --- | --- | --- | --- |
|  | →THCC | →ITR→THCC | →THCC + (→ITR→THCC) |
|  | B | B | B |
| **Patient Characteristics** |  |  |  |
| Gender (reference: male) |  |  |  |
| Female† | -0.397 | 0.004 | -0.393 |
| Age (reference: <66) |  |  |  |
| 66-70† | -0.425 | -0.122 | -0.547 |
| 71-75† | -0.753 | -0.196 | -0.949 |
| 76-80† | -0.920 | -0.236 | -1.156 |
| 81-85† | -1.381 | -0.339 | -1.720 |
| 85+† | -1.599 | -0.583 | -2.181 |
| Index year † | -0.285 | 0.015 | -0.270 |
| **Comorbidities** |  |  |  |
| CCI score (reference: 0-2) |  |  |  |
| 3 | 0.281 | -0.151 | 0.130 |
| 4† | 0.240 | -0.541 | -0.301 |
| 5+ | 0.722 | -0.683 | 0.039 |
| Prior/concurrent non-lymphoma neoplasms (reference: No) | 0.304 | -0.043 | 0.261 |
| **Complications** |  |  |  |
| Heart Disease (reference: No) † | 0.948 | 2.305 | 3.253 |
| Liver Disease† | 0.172 | 1.591 | 1.763 |
| Kidney Disease† | 1.316 | 1.518 | 2.834 |
| **Index treatment regimen** |  |  |  |
| R+/-DeVIC-based (reference: No) † | -0.046 | 2.538 | 2.492 |
| R-CHASE-based † | 0.336 | 3.705 | 4.040 |
| GDP-based without or without rituximab† | -0.117 | 1.440 | 1.323 |
| R-Treakisym-based † | 2.765 | 0.526 | 3.291 |
| R-EPOCH† | -0.137 | 2.435 | 2.298 |
| R-ESHAP-based † | 0.155 | 2.895 | 3.051 |
| ESHAP-based† | -0.453 | 3.369 | 2.917 |
| R-ICE-based † | 0.042 | 4.576 | 4.617 |
| R-DHAP-based | 0.428 | 1.919 | 2.348 |
| Other R-based† | 0.362 | 0.023 | 0.385 |
| Induction therapy before Autologous SCT regimens* | -1.266 | 1.111 | -0.155 |
| **HCRU** |  |  |  |
| Number of hospitalizations (reference: No) † | 0.123 | - | 0.123 |
| Any ICU admission† | 1.692 | - | 1.692 |
| Any PET scans† | 0.273 | - | 0.273 |
| Any MRI scans† | 0.322 | - | 0.322 |
| Any CT scans | 0.130 | - | 0.130 |
| Any emergency room visits | 0.059 | - | 0.059 |
| Any SCT† | 2.482 | - | 2.482 |
| Any radiation therapy | 0.057 | - | 0.057 |
| LOS† | 1.203 | - | 1.203 |
| Standardized Root Mean Square Residual (SRMR): 0.006‡ | | | |
| B, unstandardized coefficient; ICU, intensive care unit; ITR, (index) treatment regimen; LOS, length of hospital stays; THCC, total health care cost; USD, US dollars.  * Counted only for patients who underwent autologous SCT after the regimen; patients who underwent induction therapies but did not undergo autologous SCT after the regimen were counted as "Other chemotherapy without R"  † Standardized total effects were significant in Table 5  ‡ Hu and Bentler,1999: SRMR of <0.08 signals represent a well-fitted model | | | |

Table S6. Structural equation model of 3L cost drivers including unstandardized values

|  | Direct effect (USD) | Indirect effect (USD) | Total effect |
| --- | --- | --- | --- |
|  | →THCC | →ITR→THCC | →THCC + (→ITR→THCC) |
|  | B | B | B |
| **Patient Characteristics** |  |  |  |
| Gender (reference: male) |  |  |  |
| Female† | -0.349 | -0.023 | -0.372 |
| Age (reference: <66) |  |  |  |
| 66-70 | -0.372 | 0.045 | -0.328 |
| 71-75† | -0.703 | 0.045 | -0.658 |
| 76-80† | -1.118 | 0.044 | -1.074 |
| 81-85† | -0.993 | -0.012 | -1.004 |
| 85+† | -1.862 | -0.272 | -2.134 |
| Index year † | -0.286 | -0.003 | -0.290 |
| **Comorbidities** |  |  |  |
| CCI score (reference: 0-2) |  |  |  |
| 3 | 0.127 | -0.207 | -0.080 |
| 4 | 0.221 | -0.472 | -0.251 |
| 5+ | 0.624 | -0.611 | 0.013 |
| Prior/concurrent non-lymphoma neoplasms (reference: No) | 0.481 | -0.247 | 0.234 |
| **Complications** |  |  |  |
| Heart Disease (reference: No) † | 0.899 | 1.828 | 2.728 |
| Liver Disease† | 0.318 | 1.480 | 1.797 |
| Kidney Disease† | 1.088 | 1.490 | 2.578 |
| **Index treatment regimen** |  |  |  |
| R+/-DeVIC-based (reference: No) † | -0.003 | 1.811 | 1.809 |
| R-CHASE-based † | 0.221 | 2.869 | 3.089 |
| GDP-based without or without rituximab | -0.130 | 0.861 | 0.731 |
| R-Treakisym-based | 1.928 | -0.850 | 1.079 |
| R-EPOCH | -0.462 | 0.412 | -0.049 |
| R-ESHAP-based † | -0.047 | 1.549 | 1.502 |
| ESHAP-based | 0.064 | 0.804 | 0.868 |
| R-ICE-based † | 2.708 | 4.617 | 7.325 |
| Other R-based | 0.549 | -0.571 | -0.023 |
| Induction therapy before Autologous SCT regimens* | -0.594 | 0.390 | -0.204 |
| **HCRU** |  |  |  |
| Number of hospitalizations (reference: No) † | 0.168 | - | 0.168 |
| Any ICU admission† | 1.739 | - | 1.739 |
| Any PET scans | 0.281 | - | 0.281 |
| Any MRI scans | 0.283 | - | 0.283 |
| Any CT scans | 0.385 | - | 0.385 |
| Any emergency room visits | 0.240 | - | 0.240 |
| Any SCT† | 2.040 | - | 2.040 |
| Any radiation therapy | 0.057 | - | 0.057 |
| LOS† | 1.201 | - | 1.201 |
| Standardized Root Mean Square Residual (SRMR): 0.065‡ | | | |
| B, unstandardized coefficient; ICU, intensive care unit; ITR, (index) treatment regimen; LOS, length of hospital stays; THCC, total health care cost; USD, US dollars.  * Counted only for patients who underwent autologous SCT after the regimen; patients who underwent induction therapies but did not undergo autologous SCT after the regimen were counted as "Other chemotherapy without R"  † Standardized total effects were significant in Table 6  ‡ Hu and Bentler,1999: SRMR of <0.08 signals represent a well-fitted model | | | |
